# Floral hemp (*Cannabis sativa* L.) responses to nitrogen fertilization under field conditions in the high desert

**DOI:** 10.1371/journal.pone.0284537

**Published:** 2023-05-19

**Authors:** Mona M. Farnisa, Glenn C. Miller, Juan K. Q. Solomon, Felipe H. Barrios-Masias

**Affiliations:** 1 Department of Agriculture, Veterinary and Rangeland Sciences, University of Nevada, Reno, NV, United States of America; 2 Department of Natural Resources & Environmental Sciences, University of Nevada, Reno, NV, United States of America; CSIR-Institute of Himalayan Bioresource Technology, INDIA

## Abstract

While most studies on floral hemp (*Cannabis sativa* L.) concur that additions of nitrogen (N) increase plant growth, the performance of floral hemp is heavily influenced by environmental conditions, management and cultivar selection. In regions with a short growing season, the availability of soil N may determine plant developmental rates, final inflorescence biomass and cannabinoid concentrations, but no studies have addressed this for field-grown hemp under high-desert conditions. This field study evaluated the effect of no supplemental N and N fertilization at 90 kg ha^-1^ on three hemp cultivars (Berry Blossom, Red Bordeaux, and Tahoe Cinco) in Northern Nevada. N increased plant height, canopy cover, stem diameter and shoot biomass, but other physiological parameters were dependent on cultivar. For instance, inflorescence biomass and inflorescence-to-shoot ratio in Red Bordeaux was not affected by N fertilization. Similarly, cannabinoid concentrations were affected by timing of harvest and cultivar but not by N treatment. We evaluated the use of a SPAD meter for ease of determining leaf N deficiency, and correlations with leaf chlorophyll content showed that the SPAD meter was a reliable tool in two cultivars but not in Tahoe Cinco. N treatment increased overall CBD yield, which was driven by increases in inflorescence biomass. Tahoe Cinco was the best CBD yielding cultivar, as it maintained a high inflorescence-to-shoot ratio regardless of N treatment. Our study suggests that even though hemp may have a positive response to soil N management, adjustments based on genotype by environment interaction should be aimed at maximizing cannabinoid yield either by increasing biomass and/or CBD concentrations as long as THC levels are within the permissible <0.3% for U.S. industrial hemp cultivation.

## 1. Introduction

*Cannabis sativa* L. (hemp) is a multipurpose crop of various applications in the form of food (seed), biofuel (oil), fiber (stems), and secondary metabolites for medicinal purposes (i.e., cannabinoids) [1–4]. The most common cannabinoids are cannabidiolic acid (CBDA) and tetrahydrocannabinolic acid (THCA) although over 200 more cannabinoids exist [5]. Many of the common cannabinoids, including CBDA and THCA, are thermally converted to the commercially sold products, CBD and THC. A major restriction in floral hemp production—defined by the United States Department of Agriculture (USDA) as hemp used for the extraction of essential oils (i.e., cannabinoids) from plant resin [6]—is the total THC concentration threshold of 0.3% (the sum of THCA and THC) when grown in the U.S. [7, 8]. Hemp is an annual, dioecious crop of the Chemotype III class (i.e., high in CBD and low in THC) [9, 10], and it is mostly a photoperiodic crop, transitioning from vegetative to flowering stage at the onset of decreasing daylight and producing branches of flowers (inflorescences) on the shoot apical meristem and auxiliary meristems [11, 12]. Hemp production has increased among commercial growers across different environmental conditions in North America, but research into management practices are generally lacking. In regions where floral hemp could be adopted as an alternative crop, such as high desert environments, research on hemp responses to nitrogen (N), its monitoring under field production, and impacts on vegetative growth and cannabinoid accumulation patterns during inflorescence development is needed to prevent the loss of the crop either by late flower development or THC concentrations >0.3%.

In most crops, N availability in the soil is a crucial macronutrient for crop production and affects plant growth, development, and metabolism [13]. Differences in N requirements have been reported in hemp depending on the purpose, and research on various N fertilization levels is a key element in floral hemp production [1, 14–16]. Fiber hemp is a nitrophilic species and has been reported to respond well to N fertilization [17, 18] although some studies show conflicting results. For instance, N fertilization of 160 kg ha^-1^ increased plant matter by 25% and was more effective than 100 kg ha^-1^, but further addition of N resulted in small gains in biomass and increased plant mortality [19]. In general, 79% of N uptake occurs within the first month in fiber hemp [20] and 88% of total plant N is absorbed during the vegetative period [14, 21]. In floral hemp, N deficiency symptoms were noticed within six to seven weeks after transplanting, and N deficient plants contained 62% lower foliar N levels and 50% less biomass than plants receiving 15 mM of nitrate-nitrogen in a complete modified Hoagland solution [22]. In controlled environments, increasing N levels in a nutrient solution from 30 to 160 mg L^-1^ (ppm) increased inflorescence yield by >200% [23], but increasing N levels has also been found to decrease N use efficiency [21]. In field settings, existing levels of N in the soil make it difficult to accurately determine N requirements and fertilization strategies for hemp [24], especially in soils with high N or organic matter. The adequate amount of N fertilization to reach maximum biomass yield was found to be 120 kg ha^-1^ [24], whereas it was 240 kg ha^-1^ in another study [16]. In arid climates, such as Nevada, U.S., N limitations can often be exacerbated by drought conditions and further impact hemp N uptake, biomass and yield [25, 26]. Thus, understanding responses of floral hemp to available soil N can support growers to better fine-tune their fertilization practices.

There is a limited body of research on the responses of floral hemp to N fertilization in terms of flowering and cannabinoid production. To date, only 17 publications have evaluated nutrition management in terms of cannabinoid production and the few conducted studies provide mixed findings [27]. In a controlled environment, low (50 kg-N ha^-1^) and high (150 kg-N ha^-1^) levels of N fertilization had the highest THC concentrations compared to mid-level N fertilization (100 kg-N ha^-1^) [4]. Similarly, in a greenhouse study on medical marijuana, increasing N fertilization was found to have a negative effect on cannabinoid content [23]. Floral hemp fertilized with a nutrient solution over 150 mg-N L^-1^ (ppm) had stunted plant growth, reduced biomass and decreased cannabinoid concentrations [5]. In field settings, N application over 158 kg ha^-1^ increased biomass, but did not influence the CBD-to-THC ratio [28]; however, another study with similar N application (160 kg ha^-1^) reported an increase in CBD content and CBD yield when urease and nitrification inhibitors were used [29].

The use of Soil Plant Analysis Development (SPAD) as a proxy to determine plant N status can be a practical tool for growers to monitor plant N requirements. SPAD is a measure of ‘greenness’ and used as an indirect measure of leaf chlorophyll, allowing identification of N deficiencies quicker and without leaf destruction. Yet, correlations between SPAD, leaf chlorophyll content and leaf N content are inconsistent across the literature and only a handful of studies have examined these correlations in hemp [5, 21]. Chlorophyll fluorescence meters have become more accessible and another practical and noninvasive way to measure Photosystem II (PSII) activity and track abiotic stress or damage effects on the PSII [30–32]. These devices can support agricultural research and production by providing insight into N monitoring and management.

This study aimed to address the following objectives: (1) evaluate the response of floral hemp to N fertilization in a high desert region, (2) compare harvesting times that optimize inflorescence yield and CBD concentrations while keeping THC concentrations below 0.3%, and (3) determine the reliability of a SPAD meter in detecting N deficiencies in floral hemp. This study included three cultivars, and was conducted in Northern Nevada, U.S., which has a short growing season, and where timing for transplanting and harvesting is constrained by late or early frosts.

## 2. Materials and methods

### 2.1 Plant material and experimental setup

Three floral hemp cultivars were grown in a field (39.539944° N, -119.804244° W) at the Valley Road Experiment Station of the University of Nevada, Reno, from June to October 2021. Cultivars Berry Blossom (BB), Red Bordeaux (RB), and Tahoe Cinco (TC) were used, and obtained from Plant Fuel Genetics, LLC. Seeds were started in 72-cell seeding trays on May 22, 2021, in a 3:2 ratio of potting soil (Miracle Gro Potting Mix, OH, USA) and medium fine grain sand (Quickrete, GA, USA). Once all seedlings had emerged, seedlings were fertilized twice weekly with 2.2 ml per liter of water of 12-4-8 fertilizer (Miracle-Gro, OH, USA). After three weeks from seeding, seedlings were transplanted into the field in a randomized complete block design with four blocks total. Each block consisted of an N+ and a control treatment. Drip lines were set at four feet apart and plants were spaced three feet apart. Six drip lines conformed a block, which was split in two strips (main plots; i.e., three rows for N+ and three rows for control). Measurements were only conducted on the middle row or drip line within a treatment and the outer rows were considered as buffers. There were seven plants per bed within a plot (21 plants per plot). Two main headers were set in order to fertigate N+ treatments while irrigating control treatments (i.e., no added N) at the same time. Three rounds of 30 kg ha^-1^ N of urea (YaraVera, FL, USA) were applied through a fertigation pump (DEMA, MO, USA). Fertigations occurred on 29, 46, and 59 days after transplanting (DAT) totaling 90 kg ha^-1^ N. Hoeing and mowing were used to control weeds. Soil samples were taken from three locations in the field before fertigation. Tests indicated that organic matter was 2.89%, and ammonium, nitrate, phosphorus and potassium were 14 ppm, 32 ppm, 10 ppm, and 156 ppm, respectively.

### 2.2 Morphological measurements

The middle two most representative plants in the middle bed of the plot were marked and measured weekly for plant height and stem diameter (48 plants total). Plant height was measured from soil level to the apex of the main shoot. Stem diameter was measured 2.5 cm above soil level. Soil canopy cover images were taken every two weeks with an Agricultural Digital Camera, and pictures were pre-processed in PixelWrench2 (TETRACAM Inc., CA, USA). Images were then imported into R for analysis of canopy cover [33] as described in [34].

### 2.3 SPAD and fluorescence measurements

Starting on 45 DAT, the same 48 plants used for morphological measurements were used for SPAD and fluorescence measurements with the MultispeQ (PhotosynQ Inc., MI, USA). The most recently mature leaf, third to fifth leaf down from the top of the canopy, was marked for measurements [35]. The leaf was changed as needed between weeks of measurements as new leaves matured. Measurements were taken three times a week between 12:00 PM and 2:00 PM. Two measurements were recorded per leaf on the center leaflet avoiding the midrib. Measurements were conducted over seven weeks until 88 DAT.

### 2.4 Total leaf chlorophyll

The day the MultispeQ measurements were conducted, the same leaf was harvested for chlorophyll content (144 total samples) three times during the growing season (85, 95, and 109 DAT). Leaves were placed in a plastic bag and kept on dry ice until further processing in the laboratory, which were conducted at no or low light conditions to avoid chlorophyll degradation. Five leaf disks were bored with a number 5 cork borer for a total area of 4.64 cm^2^. Leaf disks were weighed and the disks were placed in a 2-mL Eppendorf tube (Fisher Scientific, USA) and stored at -80°C until analyzed. Upon removal from -80°C, leaf disks were crushed with a plastic pestle (Thomas Scientific), and six glass beads of a mixture of sizes ranging from 3 to 6 mm were added (Fisher Scientific, Germany). A 1 mL of 80% (v/v) acetone and water (Carolina, NC, USA) was added to the tube and vortexed for 10 minutes at speed 7 to allow the beads to further break down the leaf tissue. All tubes were placed on a shaker for 24 hours at 175–200 rpm, and then centrifuged for 2 minutes at 7000 rpm at 4°C. A 40 μl of supernatant was pipetted off into a 96-well plate (NEST Biotechnology Co., Ltd., Wuxi, China), and 160 μl of 80% acetone was added to the supernatant for a 1:4 dilution. Absorptions were measured in a spectrophotometer at 647 and 663 nm (Biotek Synergy HT Multi-Mode Microplate Reader, BioTek Instruments Inc., VT, USA). Concentrations of total chlorophyll were expressed as mg cm^-2^ and calculated based on [36] where C_t_ = 7.15A_663_ + 18.71A_647_.

### 2.5 Specific leaf area (SLA), total leaf N and δ^13^C

On the same days (85, 95, 109 DAT) the leaf was harvested for chlorophyll analysis, the leaf directly opposite to it was harvested for SLA, total leaf N and δ^13^C analyses (144 total samples). An image of each leaf was taken to calculate area with ImageJ using the Analyze Particles tool [37]. Leaves were then dried in an oven at 60°C and weights recorded after 48 hours. The same leaf samples used for SLA were then analyzed for total leaf N and δ^13^C using a Eurovector elemental analyzer (Milan, Italy) interfaced to a Micromass Isoprime stable isotope ratio mass spectrometer (Manchester, UK) in the UNR Stable Isotope Lab.

### 2.6 Flowering time and inflorescence development

The same 48 plants that were measured for all above parameters were tracked for flower initiation. A plant was considered as flowering if clusters of pistils were present at the shoot apical meristem and axillary meristems [11]. The date of flowering was recorded and weekly monitoring continued until all 48 plants reached the flowering stage.

Inflorescence samples in this study were taken twice, when pistils were at 10% and 90% of pistil dieback. The term dieback refers to the percentage of pistils that have turned from milky white to orangey brown and hardened. The 10% pistil-dieback inflorescence samples were harvested on 97 DAT for cultivar TC, 101 DAT for cultivar BB and 104 DAT for cultivar RB. The 90% pistil-dieback inflorescence samples were harvested on 111 DAT for TC and 113 DAT for BB and RB. This method of inflorescence maturity was used, instead of a specific time or date, because producers of floral hemp observe pistil dieback to determine optimal harvest time. When more than half of the plants in a plot were considered to be at 10% or 90% pistil dieback, inflorescence samples were taken from the two marked plants and composited into one sample. The top inflorescence of the shoot apical meristem and the last inflorescence of the lateral axillary branches were harvested and composite. Inflorescence samples were stored at -80°C until processed.

### 2.7 Cannabinoid analysis

From each inflorescence sample approximately a 5 g of fresh material was taken and chopped into smaller pieces (about 3 mm size) using hand shears (Vivosun, Canada). For each sample, 1 g of the chopped inflorescence material was placed into a 40 mL glass scintillation vial with 30 mL of 100% ethanol (Koptec, PA, USA) and homogenized with a hand held homogenizer (Pro Scientific, CT, USA) for 1.5 min. The solution was allowed to settle for 24 hours and 20 μL of supernatant was filtered with a plastic sterile syringe through a Nylon filter (0.45 μm, 25 mm) (Thermo Fisher Scientific, MA, USA) into a 2 mL amber glass vial (Thermo Scientific, TN, USA) with 80 μL of 100% ethanol for a 1:3 dilution. Glass vials were kept below 40°C to avoid decarboxylation before HPLC analysis.

Analytical cannabinoid standards were obtained from Restek Corporation, PA, U.S. The standards were prepared at five concentrations for CBDA (12.5, 25, 50, 100, 200 μg mL^-1^), THCA (3.125, 6.25, 12.5, 25, 50 μg mL^-1^) and combined CBD and THC (1.5625, 3.125, 6.25, 12.5, 25 μg mL^-1^) and used to contrast calibration curves. R^2^ values for linear regressions of the calibration curves of all cannabinoid standards was >0.99. High pressure liquid chromatography (HPLC) grade formic acid, methanol, and ethanol were obtained from Decon Labs (DLI 1460 Glennie circle, PA 19406). The acetonitrile (Fisher scientific, Janssen, Pharmaceuticanaan, 2440 Geel-Belgium) was of HPLC grade. Deionized water (18.4 megaohm) was obtained from an in-house water purification system. Reversed-phase chromatography was conducted using an Agilent 1100 HPLC system, with degasser, autosampler and diode-array-detector. The liquid chromatography component separation was performed on a Zorbax, SB-C18 narrow-bore column (2.1 × 150 mm, 5 μm) with a similar pre-column. The column-oven temperature was set at 25°C, and the column flow rate was set at 0.5 ml/min and solvent programmed with an initial 70:30 ratio (acetonitrile: water) to 95:5 over 10 min and held for 10 min, followed by re-establishment of the initial solvent ratio (70:30) over 1 min. Both the acetonitrile and the water contained 0.1% formic acid to maintain the carboxylic acids in the protonated form. The post-run equilibrium time was 1 min. The autosample injection volume was 15 μL. Absorbance of cannabinoids was measured at λ = 238 nm for CBDA and THCA and 220 nm for CBD and THC with a bandwidth of 4 nm. Cannabinoid concentrations were calculated as total CBD = CBD + (CBDA x 0.877) and total THC = Δ^9^-THC + (THCA x 0.877). Actual CBD and THC concentrations were usually too low to routinely measure with a negligible concentration and peak area of approximately 0.05% or less (S1 Fig).

### 2.8 Plant biomass

At 90% pistil dieback, plants were harvested for total inflorescence biomass and total shoot biomass. The two marked plants in each plot were cut at 2.5 cm above soil level. Inflorescence clusters and shoots (leaves + stem) were harvested in the plot and fresh weight recorded. A subsample of the fresh inflorescence and shoot were weighed, dried in an oven at 60°C, and dry weights recorded after 48 hours. Percent of subsample dry weights were used to calculate total dry biomass per plant.

### 2.9 Statistical analysis

All statistical analysis was performed in R 4.0.2 software [33]. The effect of ‘treatment’, ‘cultivar’, and their interaction (fixed effects) on the response variables was investigated using linear mixed-effect models (lme4 package). Random effects were chosen based on the Akaike information criteria (AIC) (ANOVA function, base R) and may have included ‘block’ or ‘plot’ with ‘DAT’ nested for response variables with repeated measures. Log and square root transformations of response variables were evaluated to find the best fit for the linear effects model. Root mean squared error was used to determine the best fit model (lmerTools package). Model assumptions for normality, homogeneity, outliers, independence, collinearity, relationships between predictors and interactions between predictors were met (performance package). For analyzing effects on time to flowering, a generalized linear mixed effects model (lme4 package) on binary data with ‘DAT’ as a repeated measure was used. The alpha for all models was 0.05. If the P-value was lower than the alpha, a post-hoc multiple-comparison procedure was conducted with the multcomp function (emmeans package). Unrestricted least significant difference (LSD) test was used to compare means in the post-hoc analysis [38]. Pearson’s correlation coefficients were analyzed to investigate the relationship between total leaf chlorophyll and specific leaf chlorophyll, SPAD, fluorescence measurements, and SLA (Hmisc and corrplot packages). All graphs were created in ggplot2 graphic package.

## 3. Results

### 3.1 Canopy cover

**Fig 1:**
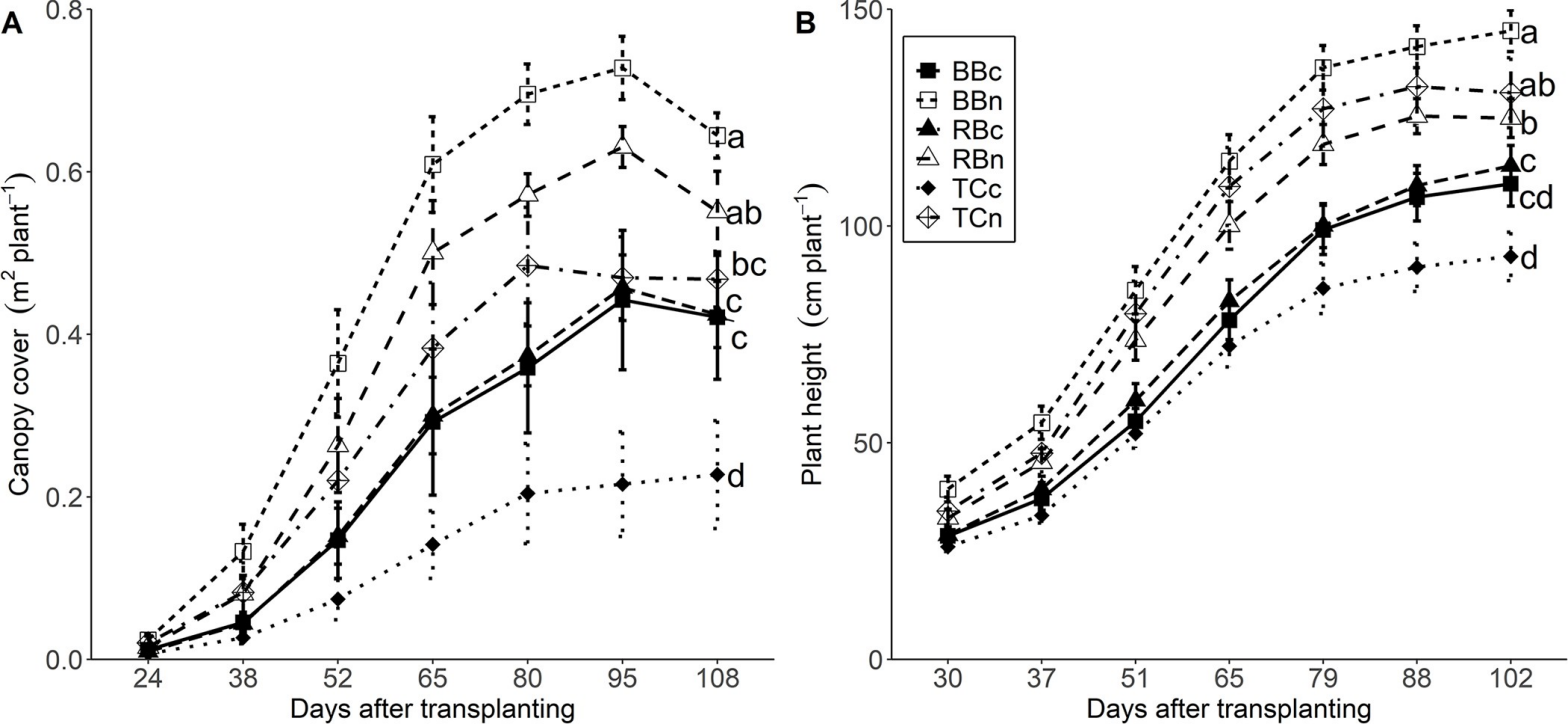
Soil canopy cover (A) and plant height (B) for N+ (n) and control (c) treatments of cultivars Berry Blossom (BB), Red Bordeaux (RB), and Tahoe Cinco (TC) grown under field conditions. Values are mean ± standard error (A and B: n = 8). Means followed by different letters are statistically different at P<0.05. (A) N treatment P<0.001, Cultivar P<0.001. (B) N treatment P<0.001, Cultivar P<0.068, N treatment x Cultivar P = 0.049.

Canopy cover was affected by N+ treatment and cultivar. The increase in canopy cover over time was higher in N+ than control treatments (Fig 1A). From 24 to 38 DAT canopy cover increased at an average of 0.6% a day in N+ treatments and 0.2% a day in controls. From 38 to 52 DAT, canopy cover increased in N+ treatments by an average of 1.3% a day compared to 0.6% a day in control treatments. The greatest increase in canopy cover for all variety and treatment combinations occurred between 52 and 65 DAT; canopy cover increased >1.8% a day for BBn and RBn, 1.3% a day for TCn, 1.1% a day for BBc and RBc, and 0.5% a day for TCc. From 65 to 80 DAT, canopy growth decreased in the N+ treatment to 0.5% a day while controls maintained growth at 0.6% a day. Maximum canopy cover was reached first by TCn at 80 DAT (0.48 m^2^ plant^-1^), while TCc was the last one to reach its maximum canopy cover at 108 DAT (0.22 m^2^ plant^-1^). By 95 DAT, BBc, BBn, RBc, and RBn reached their maximum canopy cover of 0.44, 0.73, 0.46, 0.63 m^2^ plant^-1^, respectively.

### 3.2 Plant height

Plant height was affected by N+ treatment, marginally by cultivar, and a slight interaction was found between N+ treatment and cultivar. Plant height was greater in N+ treatments over controls across all days from 30 to 102 DAT (Fig 1B). In the N+ treatment, BBn was the tallest followed by TCn and RBn. In the control treatment, BBc and RBc were similar in height, and TCc was the shortest. At 37 DAT, average height among cultivars was about 30% taller for N+ than control treatments (49 cm plant^-1^ and 37 cm plant^-1^, respectively). From 37 to 65 DAT, the N+ treatment increased height at an average rate of 2.1 cm day^-1^, compared to 1.5 cm day^-1^ for controls. After 65 DAT, gains in plant height decreased on average to 1.4 cm day^-1^ for N+ treatments and 1.2 cm day^-1^ in controls. Maximum plant height was reached first by TCn (132 cm plant^-1^) and RBn (125 cm plant^-1^) at 88 DAT. By 102 DAT, BBc, BBn, RBc, and TCc reached peak height of 109, 145, 113, and 92 cm plant^-1^, respectively.

### 3.3 Stem diameter

Stem diameter was affected by N+ treatment and cultivar (Table 1). The N+ treatment by cultivar interaction was also significant. Overall, N+ plants had greater stem diameter compared to their respective controls at each measuring date. At 37 DAT, N+ treatments had an average stem diameter of 10 mm compared to 7.5 mm in controls. The greatest increase in stem diameter occurred from 51 to 65 DAT in which N+ treatments increased by an average of 0.55 mm day^-1^ and controls by 0.39 mm day^-1^. After 79 DAT, stem diameter increased at less than 0.29 mm day^-1^ for both N+ and control plants. At 102 DAT, N+ plants reached a maximum average diameter of 31 mm compared to 22 mm in controls. Both BBn and TCn had 52% greater stem diameter than their controls. RBn had 23% greater stem diameter than RBc. BBn had the greatest stem diameter, 7% more than RBn and 26% more than TCn.

**Table 1:** Final stem diameter, inflorescence-to-shoot ratio, shoot (stem + leaves) biomass, inflorescence biomass, specific leaf area (SLA), and specific leaf N (SLN) for N+ (n) and control (c) treatments of cultivars Berry Blossom (BB), Red Bordeaux (RB), and Tahoe Cinco (TC) grown under field conditions (n = 8).

Cultivar	N Treatment	Stem Diameter		Inflorescence-to-Shoot Ratio		Dry Biomass				SLA		SLN	
						Shoot		Inflorescence					
		(mm)				(g plant^-1^)		(g plant^-1^)		(cm^2^g^-1^)		(μg cm^-2^)	
Berry Blossom	Control	22.6 ± 2.2	d	0.8 ± 0.2	bc	458 ± 134	c	330 ± 75	cd	52.4 ± 4.8	ab	865 ± 66	b
	N+	34.4 ± 2.2	a	0.4 ± 0.1	e	1344 ± 98	a	539 ± 70	ab	51.9 ± 3.8	abc	922 ± 44	ab
Red Bordeaux	Control	26.1 ± 2.5	c	0.7 ± 0.1	cd	486 ± 89	c	293 ± 41	cd	45.0 ± 4.1	c	974 ± 79	ab
	N+	32.2 ± 1.9	b	0.5 ± 0.1	de	854 ± 99	b	381 ± 12	bc	48.9 ± 5.0	bc	1020 ± 79	a
Tahoe Cinco	Control	17.9 ± 1.6	e	1.1 ± 0.2	ab	235 ± 65	d	238 ± 54	d	57.3 ± 3.9	a	821 ± 54	b
	N+	27.3 ± 1.9	b	1.3 ± 0.1	a	592 ± 117	bc	721 ± 114	a	52.0 ± 4.2	abc	958 ± 49	ab
N Treatment		P <0.001		P = 0.017		P <0.001		P <0.001		P = 0.854		P = 0.026	
Cultivar		P <0.001		P<0.001		P <0.001		P = 0.393		P = 0.001		P = 0.152	
N Treatment x Cultivar		P <0.001		P = 0.013		P = 0.036		P = 0.007		P = 0.211		P = 0.622	

Values are mean ± standard error. Means followed by different letters are statistically different at P<0.05. N treatment, cultivar and interaction effect P-values for each parameter are shown at the bottom of the table.

### 3.4 Inflorescence and shoot biomass

Inflorescence biomass was affected by N+ treatment, but not cultivar (Table 1). Yet, there was an interaction between N+ treatment and cultivar. N+ treatment had an average inflorescence biomass of 547 g plant^-1^ compared to 287 g plant^-1^ in controls. N increased inflorescence biomass in BBn and TCn by 63% and 202% compared to their controls, respectively. Nitrogen treatment did not affect inflorescence biomass in RB.

Shoot biomass was affected by N+ treatment and cultivar, and their interaction was significant (Table 1). Average shoot biomass for N+ treatments was 930 g plant^-1^ compared to 393 g plant^-1^ for controls. Shoot biomass in N+ treatments was 3-, 1.7-, 2.5-fold greater than controls for BB, RB, and TC, respectively. BBn had the greatest shoot biomass, whereas it was similar between RBn and TCn. In controls, shoot biomass in BBc and RBc were similar, while TCc had the lowest.

Inflorescence-to-shoot ratio was affected by cultivar and N+ treatment (Table 1). The N+ treatment by cultivar interaction was significant. Regardless of N treatment, TC had an inflorescence-to-shoot ratio greater than 1, and it was at least 51% higher than BB and RB. RBn and RBc had also a similar inflorescence-to-shoot ratio. Interestingly, BBc had an inflorescence-to-shoot ratio that was twice the ratio of BBn, which was mainly driven by a larger decrease in shoot than inflorescence biomass.

### 3.5 SLA and SLN

SLA was only affected by cultivar (Table 1). Cultivars BB and TC had similar SLA, on average 53 cm^2^ g^-1^, which was 12% greater than RB which had an average of 47 cm^2^ g^-1^. SLN was slightly affected by N+ treatment only, however for all cultivars N+ was not different to their respective controls (Table 1). N+ cultivars had an average SLN of 966 μg-N cm^-2^ compared to 887 μg-N cm^-2^ for control cultivars.

### 3.6 SPAD and total chlorophyll

**Fig 2:**
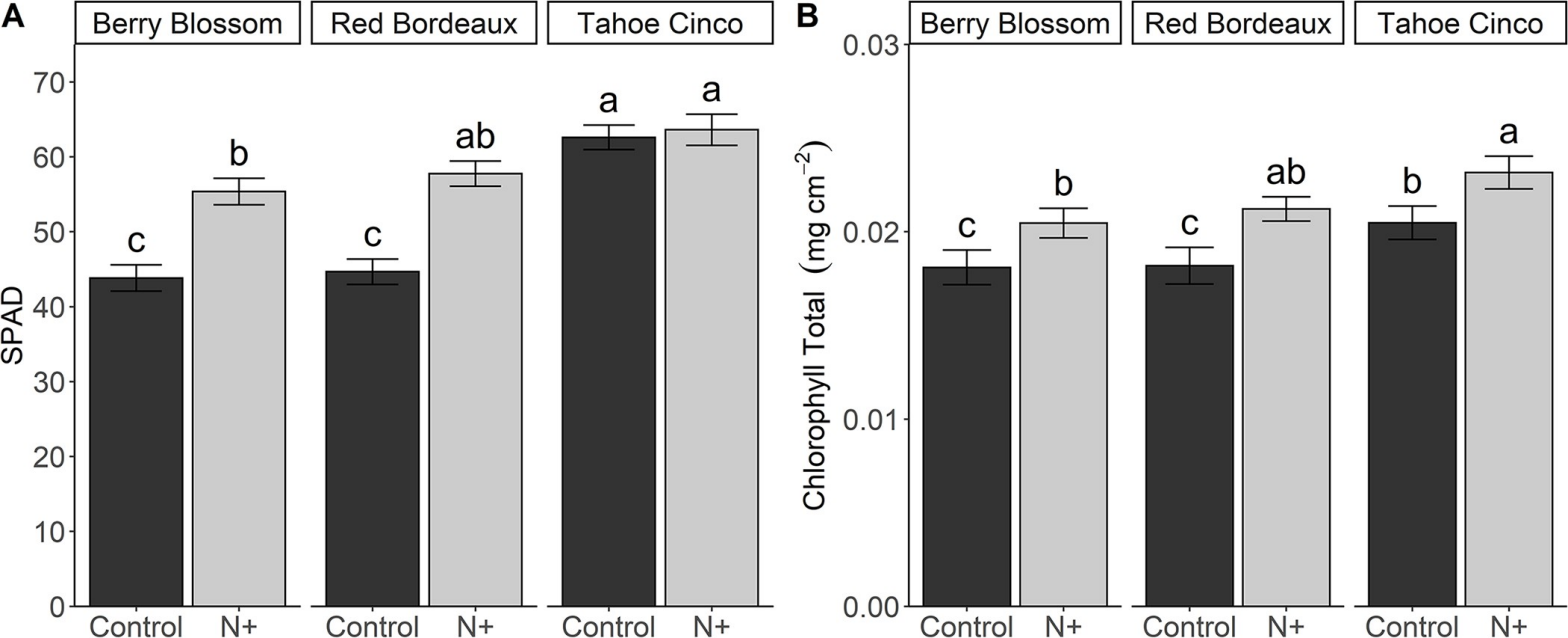
SPAD leaf values (A) and total leaf chlorophyll (B) for N+ (n) and control (c) treatments of cultivars Berry Blossom (BB), Red Bordeaux (RB), and Tahoe Cinco (TC) grown under field conditions. Values are mean ± standard error (A: n = 120; B: n = 24). Means followed by different letters are statistically different at P<0.05. (A) N treatment P<0.001, Cultivar P<0.001, N treatment x Cultivar P = 0.008. (B) N treatment P<0.001, Cultivar P<0.001.

SPAD values were affected by N treatment and cultivar, and their interaction was significant (Fig 2A). BB and RB cultivars showed a significant difference in SPAD values between N+ and controls. BBn and RBn had average SPAD values of 56, while BBc and RBc had values of 44. SPAD values for TCn and TCc did not differ. Overall, TC had the highest SPAD values among cultivars and it was on average 63, which was at least 11% greater than BBn and RBn and at least 40% greater than BBc and RBc.

Total leaf chlorophyll content was affected by N+ treatment and cultivar (Fig 2B). For all cultivars, N+ treatment increased chlorophyll content by 13%, 16%, and 13% in BBn, RBn and TCn relative to their respective controls. TC cultivar had the highest leaf chlorophyll content. TCn had an 8% higher leaf chlorophyll content than BBn. Among controls, BBc and RBc had the lowest leaf chlorophyll content of 0.018 mg cm^-2^, which was 11% lower than TCc.

### 3.7 Total leaf nitrogen and δ^13^C

**Fig 3:**
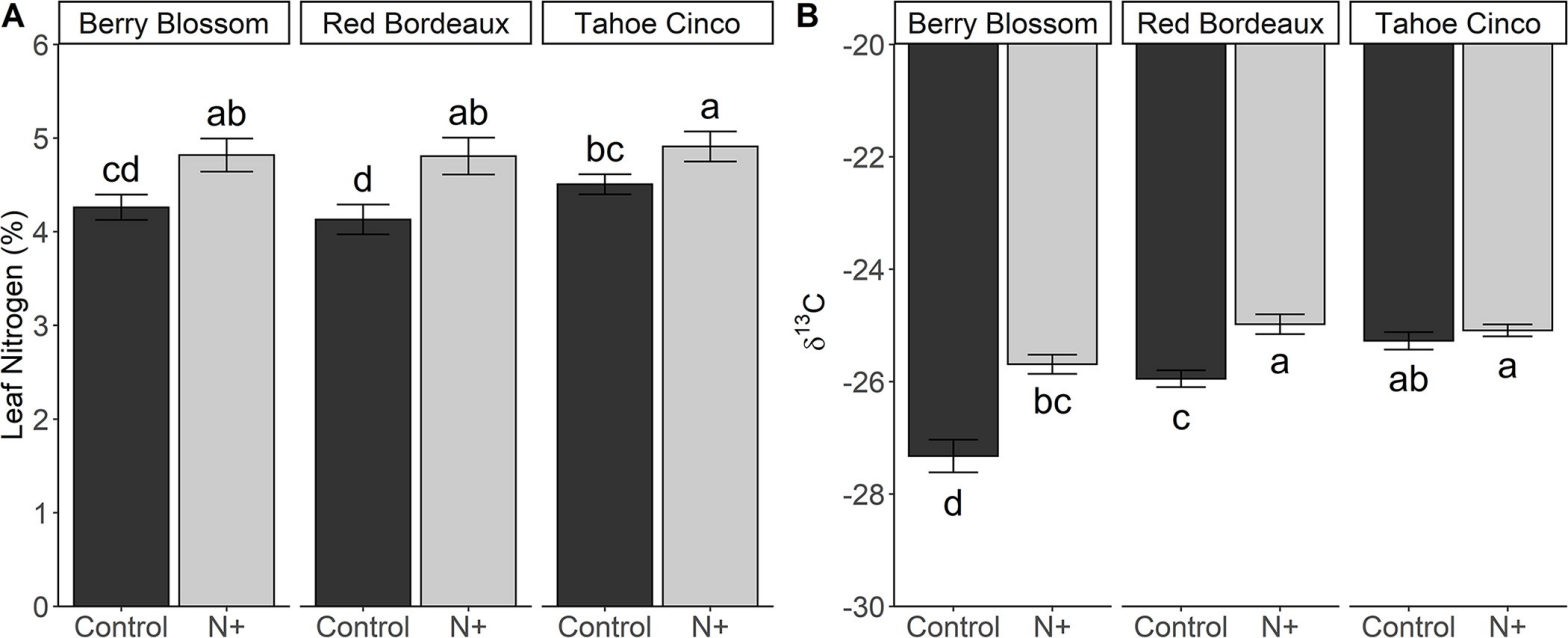
Total leaf nitrogen (A) and leaf δ^13^C (B) for N+ (n) and control (c) treatments of cultivars Berry Blossom (BB), Red Bordeaux (RB), and Tahoe Cinco (TC) grown under field conditions. Values are mean ± standard error (A: n = 24; B: n = 24). Means followed by different letters are statistically different at P<0.05. (A) N treatment P<0.001, Cultivar P<0.014. (B) N treatment P<0.001, Cultivar P<0.001, N treatment x Cultivar P<0.001.

Total leaf N was affected by N+ treatment and cultivar (Fig 3A). N+ treatments had on average a 4.8% total leaf N, which was higher than the 4.3% in controls. Unlike total leaf chlorophyll, all cultivars in the N+ treatments had similar total leaf N. BBn, RBn and TCn had 13%, 16%, and 9% more leaf N than their respective controls. TCc also had similar total leaf N content to BBn and RBn. Overall TC cultivar had the highest total leaf N content, similar to BB, but greater than RB.

δ^13^C was affected by N+ treatment and cultivar, and their interaction was significant (Fig 3B). RBn, TCc, and TCn had similar δ^13^C values of –25.1 and were the least negative. BBn and RBn had a less negative δ^13^C than their controls, which had 6.4% and 3.9% lower δ^13^C, respectively. Among controls, BBc was the most negative and was 5% and 7.5% lower than RBc and TCc, respectively.

### 3.8 Chlorophyll fluorescence parameters (Fv’/Fm’ and Φ_PSII_)

**Fig 4:**
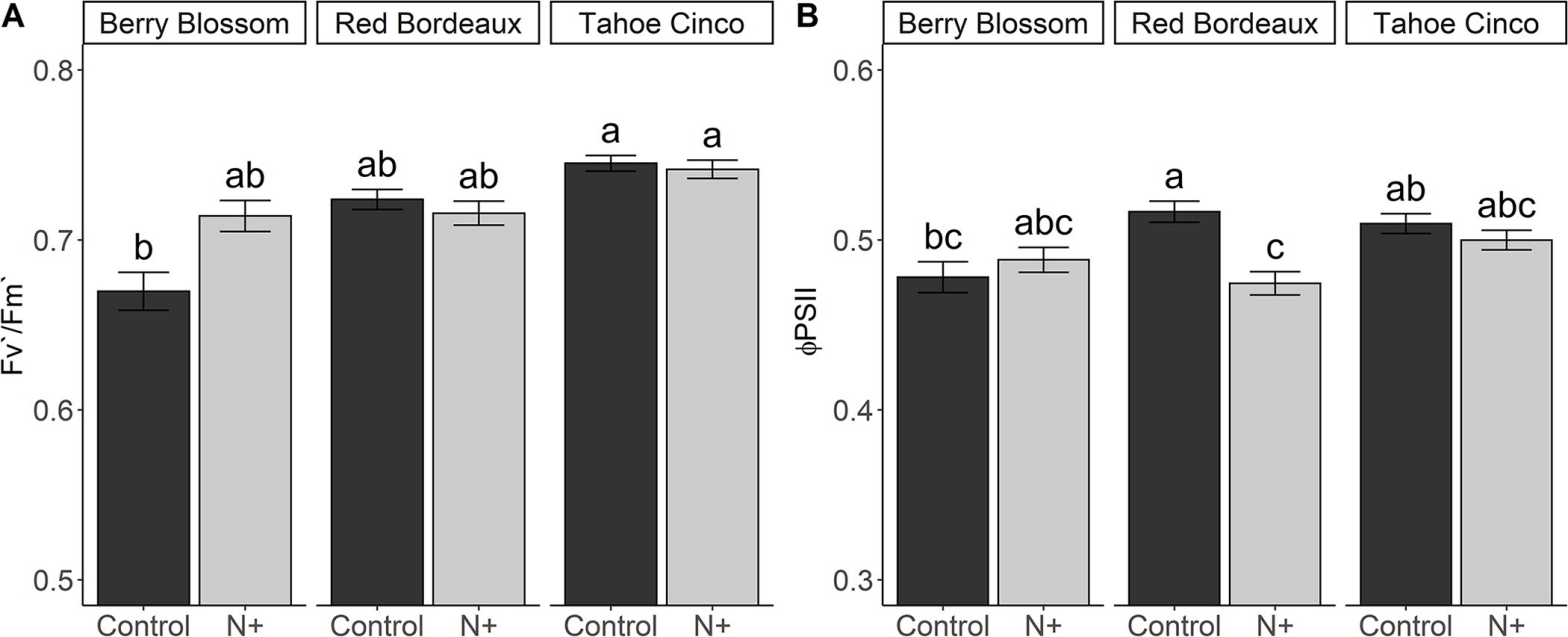
Fv’/Fm’ (A) and ΦPSII (B) leaf values for N+ (n) and control (c) treatments of cultivars Berry Blossom (BB), Red Bordeaux (RB) and Tahoe Cinco (TC) grown under field conditions. Values are mean ± standard error (A: n = 120; B: n = 120). Means followed by different letters are statistically different at P<0.05. (A) N treatment P = 0.357, Cultivar P = 0.007, N treatment x Cultivar P = 0.170. (B) N treatment, P = 0.094, Cultivar P = 0.177, N treatment x Cultivar P = 0.053.

Fv’/Fm’ was affected by cultivar only (Fig 4A). BBc and BBn were not statistically different, but BBc showed a trend of being 6% lower than BBn. TCc and TCn were similar and had the highest Fv’/Fm’, which was 11% greater than BBc, which had one of the lowest values. BBn, RBc and RBn were all similar and had an average Fv’/Fm’ of ~0.72, which was 3.4% less than the TC cultivar.

Φ_PSII_ was not affected by N+ treatment or cultivar, but both main factors showed a slight interaction (Fig 4B). RBc had the highest Φ_PSII_ value (0.52) among all cultivar and treatment combinations, while RBn had the lowest value (0.47), which was 8% lower than RBc. The Φ_PSII_ of cultivars BB and TC were not affected by treatment. BB had an average Φ_PSII_ of 0.48, and TC had an average Φ_PSII_ of 0.50.

### 3.9 Time to flowering

Time to flowering was affected by both N+ treatment and cultivar, and their interaction was significant (S2 Fig). Signs of flowering were first noticed on 59 DAT, and monitored on a weekly basis until all plants reached full flowering on 85 DAT. Overall, N+ treatment flowered consistently earlier than control treatments. TCn reached full flowering at 64 DAT; 21 days before TCc. RBn had a more gradual increase in flowering and reached full flowering at 72 DAT; 13 days before RBc. BB showed a more even pattern of flowering between the N treatments. Yet, BBn reached full flowering at 79 DAT, 6 days before BBc. On average, N+ cultivars reached full flowering approximately 17 days earlier than controls.

### 3.10 CBD and THC

**Fig 5:**
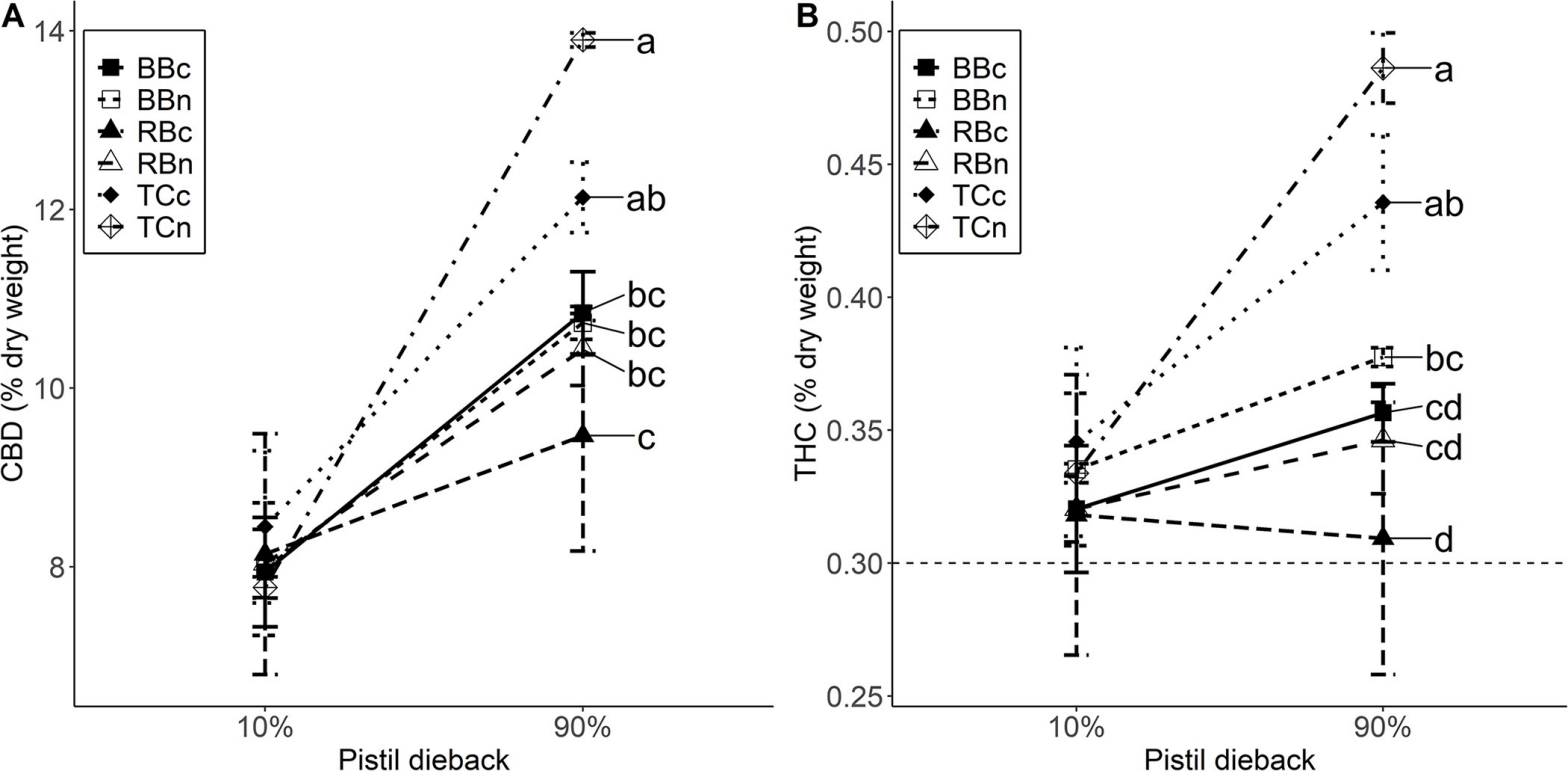
CBD concentration (A) and THC concentration (B) of inflorescence at 10% and 90% pistil dieback for N+ (n) and control (c) treatments of cultivars Berry Blossom (BB), Red Bordeaux (RB) and Tahoe Cinco (TC) grown under field conditions. Values are mean ± standard error (A and B: n = 3–4). Means at 10% pistil dieback did not significantly differ. At 90% pistil dieback, means followed by different letters are statistically different at P<0.05. Compact letter display for pistil dieback at 10% and 90% were analyzed separately (90%: a-d) (A) Pistil dieback P<0.001, N treatment P = 0.472, Cultivar P = 0.029, Pistil dieback x Cultivar P<0.001, Pistil dieback x N treatment P = 0.028, N treatment x Cultivar P = 0.881. (B) Pistil dieback P<0.001, N treatment P = 0.461, Cultivar P = 0.015, Pistil dieback x Cultivar P<0.001, Pistil dieback x N treatment P = 0.018, N treatment x Cultivar P = 0.879.

CBD concentration was affected by pistil dieback and cultivar, but not by N+ treatment (Fig 5A). Within each pistil-dieback time point, no interaction between N+ treatment and cultivar was present. Overall, CBD was higher at 90% pistil dieback compared to 10% pistil dieback. At 10% pistil dieback the average CBD concentration was 8% and did not differ among cultivars. At 90% pistil dieback TC, BB, and RB had an average CBD concentration of 13%, 11% and 10%, respectively. TCn, BBn and RBn increased in CBD by 79%, 35% and 30%, respectively, from 10% to 90% pistil dieback harvests, while TCc, BBc, and RBc increased by 44%, 37% and 16%, respectively.

THC concentration was affected by pistil dieback and cultivar, but not N+ treatment (Fig 5B). Within each pistil-dieback time point, no interaction between N+ treatment and cultivar was present. At 10% pistil dieback average THC concentration was 0.33%, and did not differ between cultivars. At 90% pistil dieback TC had the highest THC concentration of on average 0.46%, followed by BB at 0.37%, and RB at 0.33%. TCn, BBn and RBn increased in THC by 46%, 13% and 8%, respectively, from 10% to 90% pistil dieback harvests, while TCc and BBc increased by 26% and 11%, and RBc decreased by 3%.

**Fig 6:**
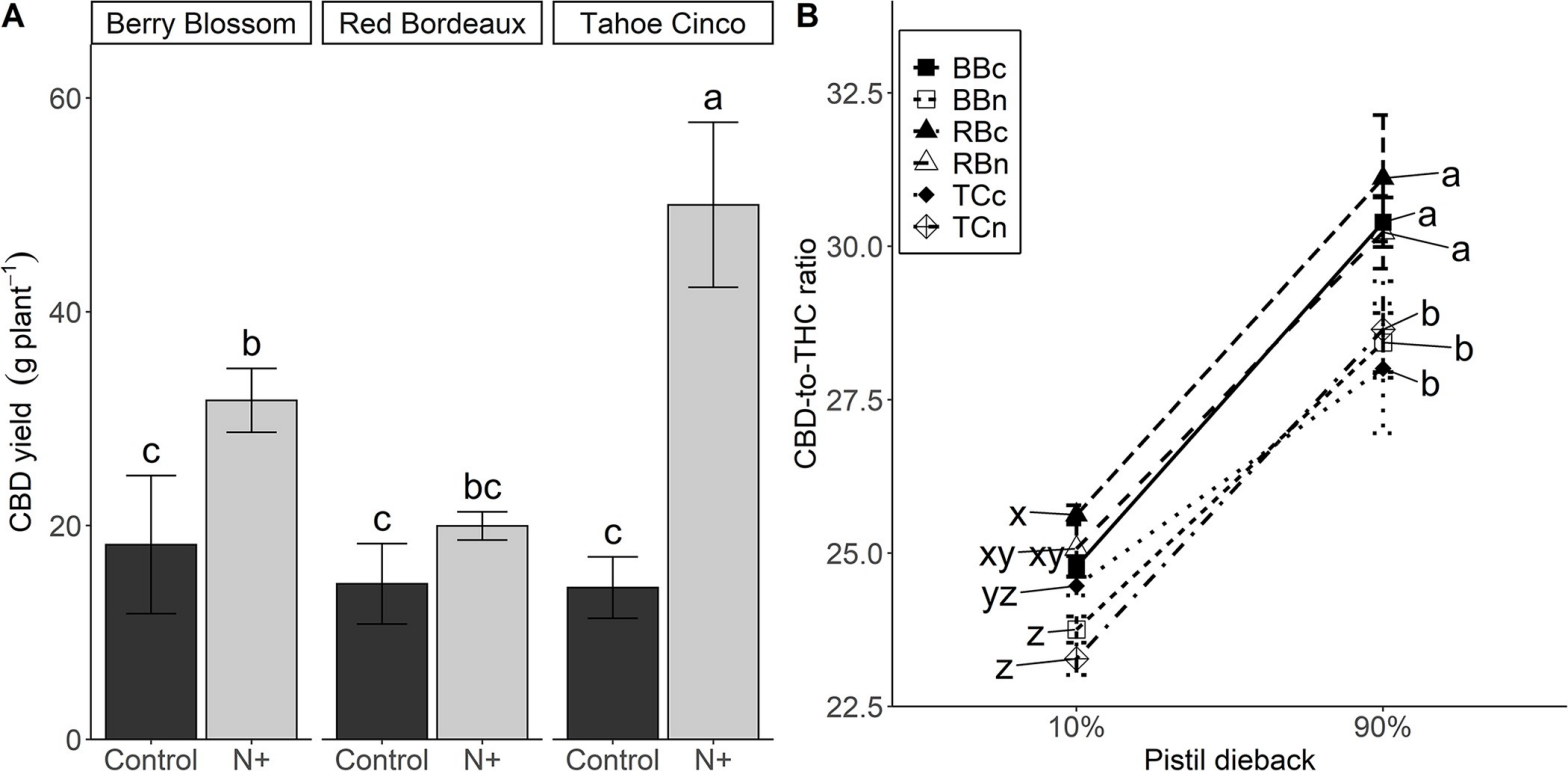
CBD yield (A) and CBD-to-THC ratio (B) at 10% and 90% pistil dieback for N+ (n) and control (c) treatments of cultivars Berry Blossom (BB), Red Bordeaux (RB) and Tahoe Cinco (TC) grown under field conditions. Values are mean ± standard error (A and B: n = 3–4). Means followed by different letters are statistically different at P<0.05. Compact letter display for pistil dieback at 10% and 90% were analyzed separately (10%: x-z; 90%: a-d). (A) N treatment P<0.001, Cultivar P = 0.002, N treatment x Cultivar P = 0.002. (B) Pistil dieback P<0.001, N treatment P = 0.001, Cultivar P<0.001, N Treatment x Cultivar P = 0.053, Pistil dieback x N Treatment P = 0.592, Pistil dieback x Cultivar P = 0.357.

CBD yield, based on biomass and CBD concentration, was affected by N+ treatment and cultivar, and their interaction was significant (Fig 6A). N+ treatments had an average CBD yield of 34 g plant^-1^ compared to 16 g plant^-1^ in controls. TCn had the highest CBD yield, and it was 252% greater than TCc. BBn had the second highest CBD yield, and it was 74% greater than BBc. There was no difference in CBD yield per plant between the N treatments of RB. Among N+ treatments, TCn had 150% and 58% greater CBD yield than BBn and RBn, respectively. There was no difference in CBD yield among controls.

The CBD-to-THC ratio was affected by pistil dieback, N+ treatment, and cultivar; and a slight N treatment by cultivar interaction was present (Fig 6B). Overall, cannabinoid ratio increased from 10% to 90% pistil dieback from an average of 24.5 to 29.5. The cannabinoid ratio increased by 23% for BBc and TCn, 21% for RBc and RBn, 20% for BBn, and 14% for TCc. At 90% pistil dieback TC had the lowest average ratio of 28.3, followed by BB at 29.4, and RB at 30.7.

### 3.11 Correlations

**Fig 7:**
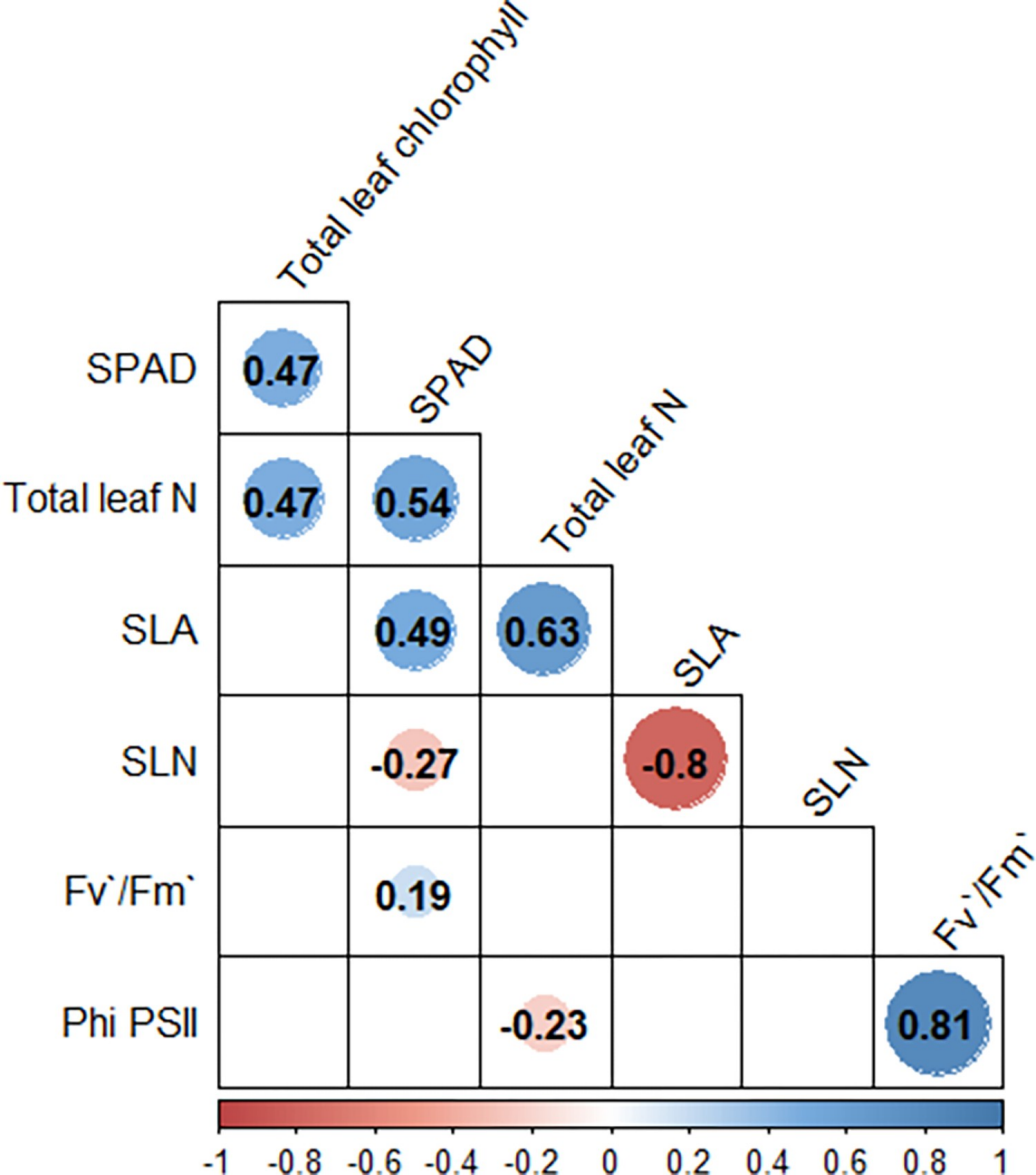
Correlation between SPAD, leaf chlorophyll content, leaf N content, SLA, SLN, Fv’/Fm’, and Φ_PSII_.

A Pearson correlation matrix was conducted in this study to determine the relationships between SPAD values, total leaf chlorophyll, total leaf N, SLN, SLA, Fv’/Fm’ and Φ_PSII_ (Fig 7). The highest positive correlations were found between the two chlorophyll fluorescence parameters: Fv’/Fm’ and Φ_PSII_ (r = 0.81). SLA had a high negative correlation to SLN (r = -0.80), and a positive correlation to total leaf N (r = 0.63). SPAD readings had positive correlations with total leaf chlorophyll, total leaf N, and SLA (r = 0.47, 0.54 and 0.49, respectively).

## 4. Discussion

Although the effect of N on crops is well documented, studies on hemp have not shown a clear response to N fertilization [5, 21, 39]. In this study, N fertilization at a rate of 90 kg ha^-1^ was found to be important for morphological and physiological development of floral hemp, especially early in the growing season. Inflorescence and shoot biomass, time to flowering, and CBD yield were all positively influenced by N fertilization. Leaf chlorophyll, leaf N concentration and SPAD values increased due to N fertilization and were correlated within each other, but cultivars responded differently, suggesting that the use of instruments based on ‘greenness’ should be calibrated to particular production conditions. The effect of N fertilization on PSII was less evident and showed significant interactions with fertilization treatment and cultivar. CBD and THC concentrations were found to be more dependent on cultivar and pistil dieback (i.e., inflorescence maturity) than N fertilization. The cultivar by N treatment interaction affected overall CBD yield based on inflorescence biomass and suggests that cultivar is an important consideration when growers are looking to increase overall CBD yield per unit area while maintaining THC below 0.3%.

### 4.1 Morphological growth and biomass

Nitrogen fertilization accelerated plant development early in the growing season, which can provide advantages in regions with a short growing season. For instance, at 38 DAT, N+ plants had an average canopy cover more than twice that of the control plants, which supports other literature that floral hemp responds to early N supply [14]. Ranges of N used in hemp research vary from 60 to 240 kg ha^-1^, however levels of N fertilization beyond 150 kg ha-1 N had no effect on increasing yields [24]. It has been suggested that slow release fertilizers are preferred in drought prone semiarid environments to facilitate N uptake [29]; yet, our preliminary data have not shown a clear response under conditions in Northern Nevada. Overall, plant architecture was dependent on cultivar and its response to soil N availability, which affected total plant and inflorescence biomass. N fertilization can increase the quantity of axils and nodes, and favor inflorescence production [11, 40]. For instance, plant height was positively correlated with inflorescence biomass [5], but in this study it was only observed for cultivars BB and TC. In addition, plant size based on soil canopy cover was not a good indicator of final inflorescence biomass for one of the cultivars (i.e., TC). The average inflorescence yield for day-length-neutral cultivars is approximately 450 g plant^-1^ [41]. The present study found that N fertilization increased inflorescence biomass by 1.5 to 3.0 fold compared to control treatments in cultivars BB and TC (209 and 483 g plant^-1^, respectively), which emphasizes the need for identifying an optimal N fertilization strategy based on soil and cultivars. The reduced soil N availability of the control treatment tended to increase the inflorescence-to-shoot ratio in BB and RB compared to their N+ counterparts. This likely resulted from lower allocation of resources into vegetative growth (e.g., stem biomass) and a lower impact of N deficiency in inflorescence development [16]. On the other hand, TC cultivar had an overall higher inflorescence-to-shoot ratio, indicating an inherit characteristic less dependent on soil N availability.

Positive correlations have been reported between leaf N content, inflorescence biomass, and CBD yield in floral hemp [42, 43]. The increased inflorescence biomass from N fertilization resulted in increased CBD yield for TCn and BBn compared to controls. In addition, the higher CBD concentration in TCn resulted in a 1.6-fold increase in CBD yield when compared to BBn; although TCn and BBn had similar inflorescence biomass. In this study, cultivars with higher inflorescence-to-shoot ratio may be better suited for growers as the potential for higher yields increases even when soil N availability is low or affected by other factors such as drought.

### 4.2 Time to flowering

Flowering initiation was earlier in N fertilized RB and TC, but not in BB; yet, the start of flowering is a critical factor for timing of harvest, inflorescence yield, and cannabinoid concentration [10, 44]. An evaluation of 30 floral hemp cultivars showed great variation among and within cultivars for flowering time [10]. In this study, all cultivars exhibited very different flowering patterns. In the control treatment, flowering was not consistent across all individuals and was protracted over a period of 15 days, while N+ treatments had a faster and more consistent flowering pattern. The emergence of auxiliary female flowers and the initiation of terminal flowering at shoot apices has a low correlation for determining the timing to full flowering [10]. This may explain why control plants with a delayed vegetative development (e.g., canopy cover and plant height) exhibited auxiliary inflorescences and pistils early in the growing season, but a delayed time to reach terminal flowering in the shoot apical meristems. Other studies have not shown earlier flowering with increasing supplementation of N under controlled environments [4]. Environmental factors affecting flowering are high temperature and longer day length, which have been reported to induce earlier flowering but longer developmental time [4]. In this study, flowering started in August with an average day length of 13.7 hours and daytime temperature of ~30°C. For growers under similar growing conditions, the identification of cultivars with earlier flowering and a short flowering period may be beneficial. For instance, the high inflorescence biomass in TCn may be related to its early and short flowering period (5 days). Early flowering genotypes have longer accumulation time for inflorescence biomass [19, 45], especially under high desert conditions where low night temperatures and the increased likelihood of frost may decrease inflorescence quality [46].

### 4.3 Cannabinoid content

Our study found that N fertilization did not affect CBD and THC concentrations, which adds to several studies reporting conflicting results about the effects of N on secondary metabolites [5, 23, 29]. Some studies have reported similar findings that the main effect of N fertilization is on CBD yield per plant driven by increased inflorescence biomass [28, 42, 43]. Stress and varying environmental conditions have been shown to affect cannabinoid concentrations in floral hemp [4, 41]. Previous studies have found that high temperatures, long hours of daylight, high humidity, and altitude could increase CBD and THC concentrations in hemp [47–49]. Drought stress has been found to decrease CBD and THC concentrations in floral hemp [50]. In our study, in addition to the N treatment, altitude, daily high temperature fluctuation, low relative humidity and wildfire smoke were environmental variables that could have affected cannabinoid concentrations. Yet, cultivar BB has shown consistency in cannabinoid concentrations in this and other studies conducted under different environmental conditions [5].

Decreases in secondary metabolite production, due to high N input, may be due to resource allocation into chlorophyll and other N metabolite processes (e.g., biomass) as opposed to cannabinoid production—which does not contain N [23]. This might explain why we found similar CBD and THC concentrations in N+ and control plants, but faster growth and total biomass in N+ plants. Our findings suggest that tradeoffs between biomass and cannabinoid concentrations can determine total yield per unit area based on the commercial purpose for floral hemp (e.g., medicinal or recreational).

Harvesting time, as determined by pistil dieback, increased the concentration of CBD and THC, which has been shown to increase linearly [10] as flowering progresses [51, 52]. In the high desert, timing of harvest is crucial as early frosts can damage the crop. In our study flower development to 10% pistil dieback took approximately seven weeks from initial flowering, and from 10% to 90% pistil dieback only two weeks, which is a short time window in which cannabinoid concentrations increased by up to 79% for CBD and 46% for THC in cultivar TC.

Conflicting information exists on the pace of THC accumulation in floral hemp, and reported concentrations may depend on the sampling protocol. For instance, in this study inflorescence sampling for cannabinoids was done in very small quantities from the inflorescence without including any stems, which is included in sampling by the Nevada Department of Agriculture (e.g., 10 inflorescences of at least 6” long). Although our THC concentrations were above 0.3%, the official testing for compliance showed that the entire study was within the required THC concentration. In addition, the location of the inflorescence in the plant affects cannabinoid concentrations, with top inflorescences usually containing more CBD and THC than in lateral branches [23, 53]. Stage of development or maturity also affects THC concentrations as studies found that as early as one week from terminal flower initiation [10] and between four and seven weeks from initial flowering [5, 41] cultivars may surpass the 0.3% THC threshold required by U.S. regulations.

As cannabinoid concentrations changed during inflorescence development and maturation, changes in CBD-to-THC ratio increased, which can help maintain hemp in compliance with USDA regulations. At 10% pistil dieback, the CBD-to-THC ratio across all samples was 24.5, which is similar to a study under controlled environment [46], however at 90% pistil dieback our cannabinoid ratio increased to 29.2. Overall, CBD-to-THC ratios vary and are influenced by hemp chemotypes [10], cultivars, day-length sensitivity [4], and secondary metabolite formation of CBD and THC from initial flowering to senescence and harvest. Chemotype III plants usually have a CBD-to-THC ratio of 21:1, but can vary based on genotype and timing of sampling from 20:1 to 30:1 [10]. As breeders continue to develop strains that can maximize CBD while maintaining THC below the 0.3% threshold, environmental stressors (i.e. temperature, drought) that affect their accumulation in the inflorescence can be monitored to achieve target yields.

### 4.4 Leaf parameters

Nitrogen fertilization increased chlorophyll and N content in leaves, which is an expected response in most crops, including hemp, to additions of N when compared to a control treatment [4, 21, 54]. Generally, TC had higher chlorophyll levels in both the N+ and controls indicating a higher allocation of N to chlorophyll than the other two cultivars even though leaf N content was less variable among all cultivars. The allocation of leaf N to chlorophyll depends on species and environment and has been reported to change as a result of N fertilization [55]. A report on >100 floral hemp CBD cultivars and >6,000 hemp plant tissue samples in field and greenhouse conditions in North Carolina found that foliar N concentrations ranged from 3.3%– 5% [35]. Other studies have reported similar ranges [56, 57]; however, [25] reported a lower range of 2.7% to 4.5% N. In our study, all cultivars had similar leaf N, between 4–5%, which is twice as much as reported by [58] even though both studies used similar fertilization rates. One explanation may be the choice of cultivars and a high potentially mineralizable N, from organic matter, in our study site.

SPAD readings, as a practical approach to monitor N content, showed a better correlation with leaf N than with chlorophyll content, and it was influenced by cultivar. Previous studies have highlighted the intra-species variation between SPAD readings and leaf chlorophyll content [59, 60]. Overall, SPAD and leaf N tend to have a linear correlation, while SPAD and leaf chlorophyll content tend to correlate non-linearly [55, 61]. This may help explain why TC showed no difference in SPAD readings between N+ and control plants, had an overall high SPAD reading, and a lower correlation with chlorophyll content. Non-uniform distribution of chlorophyll in leaves can result in chlorophyll meters inaccurately calculating values as a result of a sieve or detour effect [55, 59]. In rice, low N treatment had slightly lower but not different SPAD values to the medium and high N treatments, which was attributed to a high coverage of chloroplasts in the rice mesophyll-cell periphery that led to minimal changes in SPAD readings in the low N level [55]. In addition, environmental factors such as light conditions and light-dependent chloroplast movement can affect SPAD readings, especially under low N fertilization [55].

Our data suggests that SPAD readings below 46 for floral hemp are indicative of low leaf chlorophyll and N content, and should be considered as N deficiency, which is in line with the SPAD threshold of 44 reported by [5]. Cultivars like TC that do not reflect N deficiency through a SPAD reading can be harder to monitor and other approaches may be needed (e.g., petiole N).

Few studies have documented fluorescence parameters in hemp to any type of nutrient stress. Our study suggests that even though N had an effect on leaf N and chlorophyll content, there were no changes in the fluorescence parameters evaluated under field conditions. One study has shown that PSII activity increased in hemp fertilized with N [62]. The only change observed in this study was the Φ_PSII_ in RBn being lower than RBc, which can indicate lower capacity for electron transport rate and more PSII reaction centers closed. This is indicative of damage to the photosynthetic electron transport chain causing photoinhibition and oxidative damage to membranes [63]. In addition, higher δ^13^C (less negative) in the N treatment suggests that a higher photosynthetic capacity due to increased chlorophyll could have resulted in unsustainable transpiration rates that led to daily, transient drought stress and closing of stomata. Further studies on gas exchange would be needed to understand patterns of diurnal stomatal conductance and photosynthetic rates. In addition, the leaves used for foliar δ^13^C analyses were harvested during prolonged periods of wildfire smoke and poor air quality. O^3^ has been reported to inhibit PSII activity, reduce stomatal conductance, and decrease photosynthetic rate [64].

## 5. Conclusion

This research adds to the growing body of literature on field floral hemp and the effects of N fertilization on cannabinoid accumulation, leaf N and PSII functioning. Plant physiological growth and time to flowering were positively influenced by N fertilization. CBD yield, cannabinoid concentrations and the CBD-to-THC ratio were not as dependent on N fertilizations, but were more affected by cultivar and timing of harvest. The reliability of SPAD measurements to detect N deficiencies depended on cultivar, however, positive correlations were found between leaf chlorophyll content, leaf N content, and SPAD readings. Fluorescence measurements gave mixed results and additional research is required into using these tools to understand crop performance and detect N deficiencies in field floral hemp. Overall, the choice of cultivar based on cannabinoid concentration and accumulation overtime coupled with total inflorescence biomass were drivers of CBD yield in this study.

## Supporting information

S1 Fig Output of HPLC chromatogram at 220 wavelength indicating peaks and retention times of CBDA, CBD, THC, and THCA. (TIF)

S2 Fig Flowering time indicated as days after transplanting for control (dark grey) and N+ (light grey) treatments of cultivars Berry Blossom (BB), Red Bordeaux (RB) and Tahoe Cinco (TC) grown under field conditions. (TIF)
