# Supplementary figures and images for: Floral hemp (Cannabis sativa L.) responses to nitrogen fertilization under field conditions in the high desert
Source: PLoS One. 2023 May 19;18(5):e0284537. doi: 10.1371/journal.pone.0284537 (PMC10198490; doi:10.1371/journal.pone.0284537)

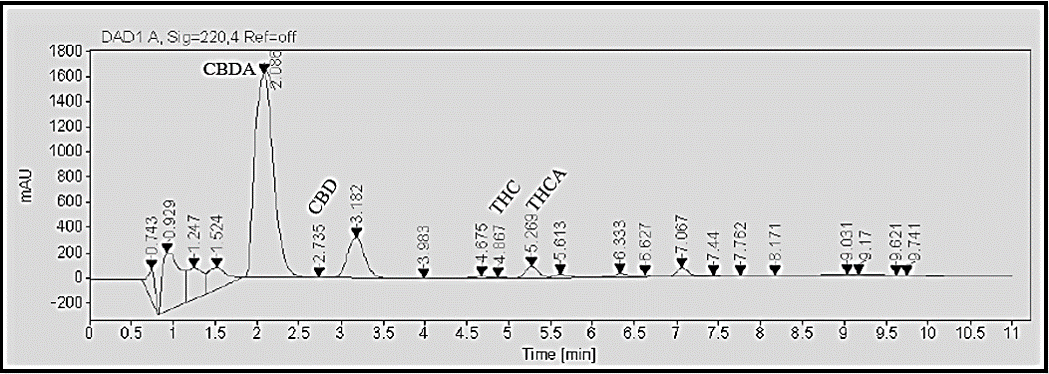

Supplement: S1 Fig — (TIF) [file pone.0284537.s001.tif]

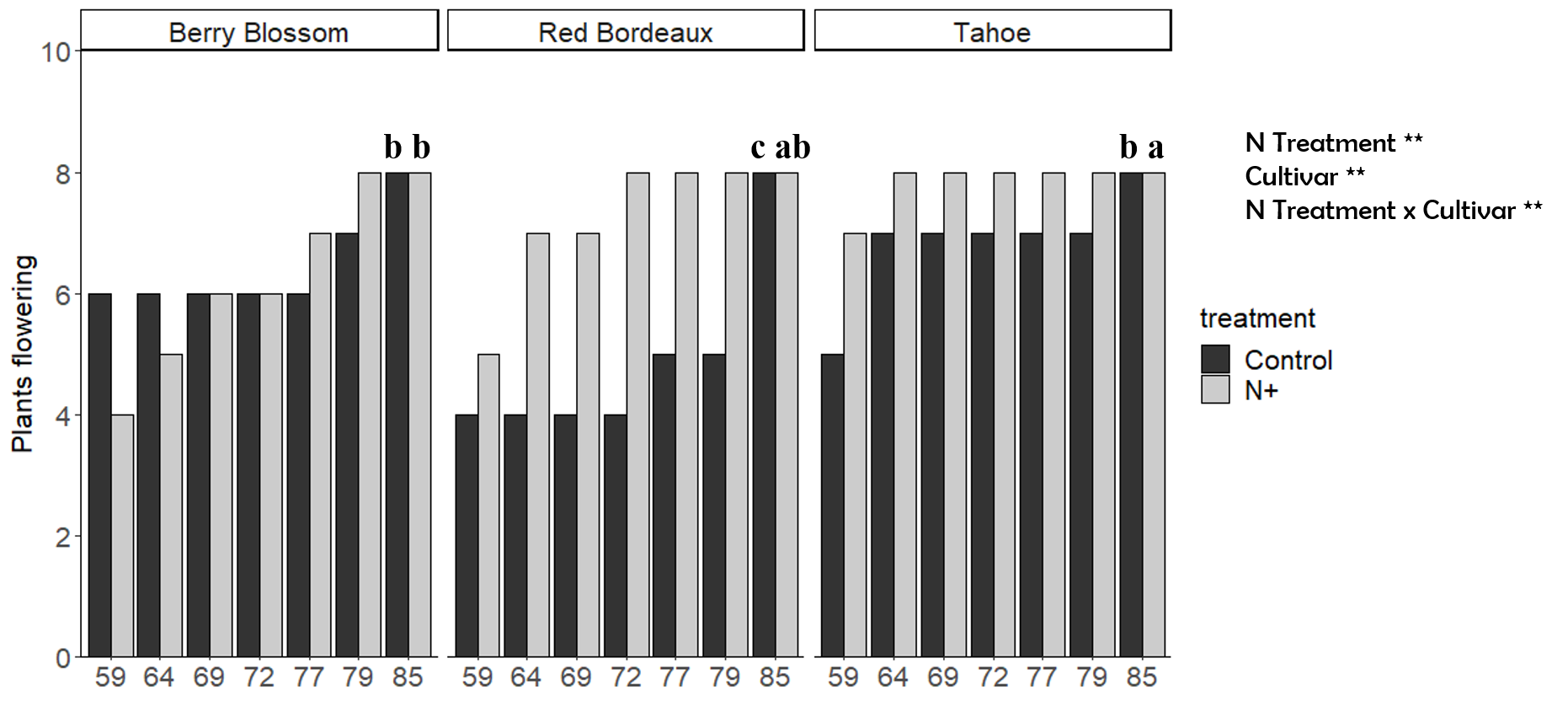

Supplement: S2 Fig — (TIF) [file pone.0284537.s002.tif]
